# Gate engineering Fabry-Pérot resonance in altermagnetic junctions

**DOI:** 10.1038/s41598-025-29607-w

**Published:** 2025-11-27

**Authors:** Qianqian Lv, Yong Xu, Jun-Feng Liu, Pei-Hao Fu, Xiang-Long Yu

**Affiliations:** 1 School of Humanities and Basic Sciences, Shenzhen University of Information Technology, Shenzhen, 518172 China; 2 Institute of Materials, Ningbo University of Technology, Ningbo, 315016 China; 3 School of Physics and Materials Science, Guangzhou University, Guangzhou, 510006 China; 4 School of Science, Sun Yat-sen University, Shenzhen, 518107 China

**Keywords:** Altermagnetism, Spin filtering, Fabry-Pérot resonance, Gate control, Materials science, Nanoscience and technology, Physics

## Abstract

Electrically generating and controlling spin-polarized transport is a central objective in spintronics. Altermagnets, which exhibit compensated magnetic order and symmetry-protected spin splitting, offer a promising route toward this goal without requiring net magnetization, magnetic fields, or spin-orbit coupling. Here, we investigate coherent spin transport in a two-dimensional *d*-wave altermagnetic junction connected to normal metal leads. The anisotropic exchange field induces spin-dependent effective masses, resulting in distinct Fabry-Pérot resonance conditions for spin-up and spin-down electrons. When the junction length matches half-integer multiples of the spin-dependent wavelength, one spin channel is resonantly transmitted while the other is suppressed, yielding fully spin-polarized transport. Employing the quantum scattering formalism, we show that the spin polarization of the transmitted current can be controlled by tuning (i) the gate potential in the altermagnet, (ii) the interfacial barrier strength, and (iii) the orientation of the altermagnetic field. These control parameters enable electrically tunable spin filtering and provide a diagnostic to distinguish between distinct *d*-wave altermagnetic symmetries. In particular, we show that the $d_{x^2 - y^2}$-wave altermagnet supports robust gate-controlled spin-polarized current even in the high-barrier tunneling regime, a behavior absent in its $d_{xy}$-wave counterpart. Our results establish a field-free, gate-controlled mechanism for spintronic functionality rooted in the crystalline anisotropy of altermagnetic materials.

## Introduction

Spintronics, which exploits the spin degree of freedom of electrons for information processing, holds promise for next-generation devices combining high speed, low energy consumption, and nonvolatility^1–5^. Most spintronic functionalities, such as giant magnetoresistance^6,7^, spin-transfer torques^8^, and spin filtering^2,3^, rely on magnetic materials. Ferromagnets offer robust spin polarization due to their net magnetization^1,2^, whereas antiferromagnets provide ultrafast spin dynamics and resilience against external magnetic fields^3–5^. Conventional spintronic architectures typically depend on magnetic fields or magnetization dynamics to manipulate spin degrees of freedom, which limits scalability due to challenges in local control, energy efficiency, and device miniaturization^1^.

A promising alternative lies in electrically controlled spintronics^9^, where spin transport is modulated via electrostatic gating. Such gate-controlled mechanisms enable fast, localized, and low-power spin logic that is readily integrable with existing semiconductor platforms^10,11^. However, realizing spintronic devices that operate entirely under electrical control, without relying on magnetic fields or strong spin–orbit coupling^9^, remains an outstanding challenge. Achieving this goal within magnetic systems that lack net magnetization and relativistic effects would mark a significant advance toward field-free and scalable spintronic technologies.

Recently, unconventional magnetic materials have emerged as a novel class of systems with nontrivial symmetry properties and spin functionalities^12–22^. Among them, altermagnets constitute a distinctive subclass characterized by compensated magnetic order that breaks time-reversal symmetry while preserving zero net magnetization. Due to their crystalline symmetry, AMs support momentum-dependent, nonrelativistic spin splitting even in the absence of spin-orbit coupling, making them attractive candidates for field-free, gate-tunable spintronics. Unconventional magnets can be categorized by the angular momentum character of their exchange fields into *p*-, *d*-, *f*-, *g*-, and *i*-wave types^18–20,23–29^. In particular, altermagnets have been experimentally identified in candidate materials such as MnTe^30,31^, Mn$_5$Si$_3$^32,33^, and CrSb^34^. Among them, RuO$_2$ remains under active debate, with transport evidence for symmetry-breaking responses but no conclusive momentum-resolved spin splitting^35–39^. These systems exhibit a broad range of spin-dependent responses, including giant magnetoresistance^40^, anomalous Hall effects^12,31,32,36,38^, spin-orbit torques^41–43^, spin filtering effects^16,17,44–46^, strongly correlations in Mott insulators^47–49^, non-linear transport^27^, non-Hermitian effects^50,51^, spin pumping^52^, nonlinear transport^27^, light-induced spin density and spin control^53–55^, multipoles^56^ among other phenomena^13–16,21,22^. Additionally, AMs have been proposed as superconducting spintronic platforms supporting parity-sensitive Cooper pairs^55,57–61^, orientation-tunable Josephson junctions^62–66^, and nonreciprocal superconducting diodes^67–69^. Furthermore, altermagnet magnets have been used for realizing Majorana corner modes in higher-order topological superconductors without external magnetic fields^61,70–78^. Despite these advances, including recent proposals for spin filtering and light-induced control^45,55^, the exploitation of altermagnetic anisotropy for achieving gate-tunable spin transport in normal-state devices remains in its infancy. In particular, whether the intrinsic momentum-space structure of AMs can support coherent, electrically controlled spintronic functionalities without requiring magnetic fields or relativistic effects remains an open question.

**Fig. 1:**
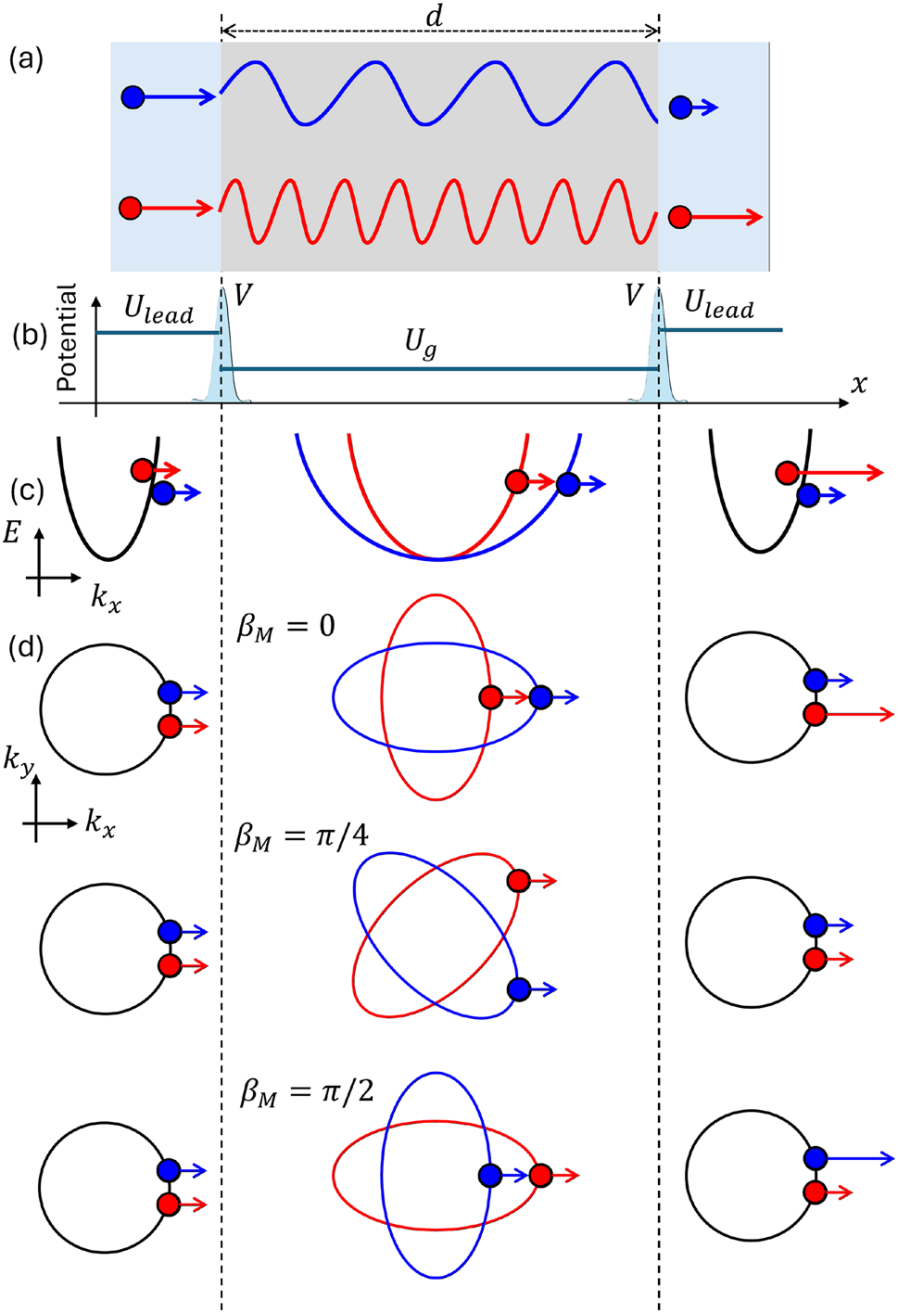
Schematic illustration of spin-selective Fabry–Pérot interference in an altermagnetic junction. (a) A two-terminal device composed of a *d*-wave altermagnet (gray) connected to normal metal leads (light blue). Spin-up (blue) and spin-down (red) electrons acquire different phases in the altermagnetic region due to spin-dependent wavevectors $k^\sigma$, resulting in distinct resonance conditions and spin-polarized transmission. (b) Electrostatic potential profile of the junction: $U_L$ in the leads, $U_g$ in the central altermagnet of length *d*, and interface barrier *V* at the boundaries. (c) Schematic band structure in the leads and the altermagnetic region for $\beta _M = 0$, showing spin splitting in the transport direction. (d) Spin-resolved Fermi surfaces and propagation directions in the $k_x$–$k_y$ plane for $\beta _M = 0$, $\pi /4$, and $\pi /2$, illustrating the anisotropic spin splitting of the *d*-wave altermagnet.

**Fig. 2:**
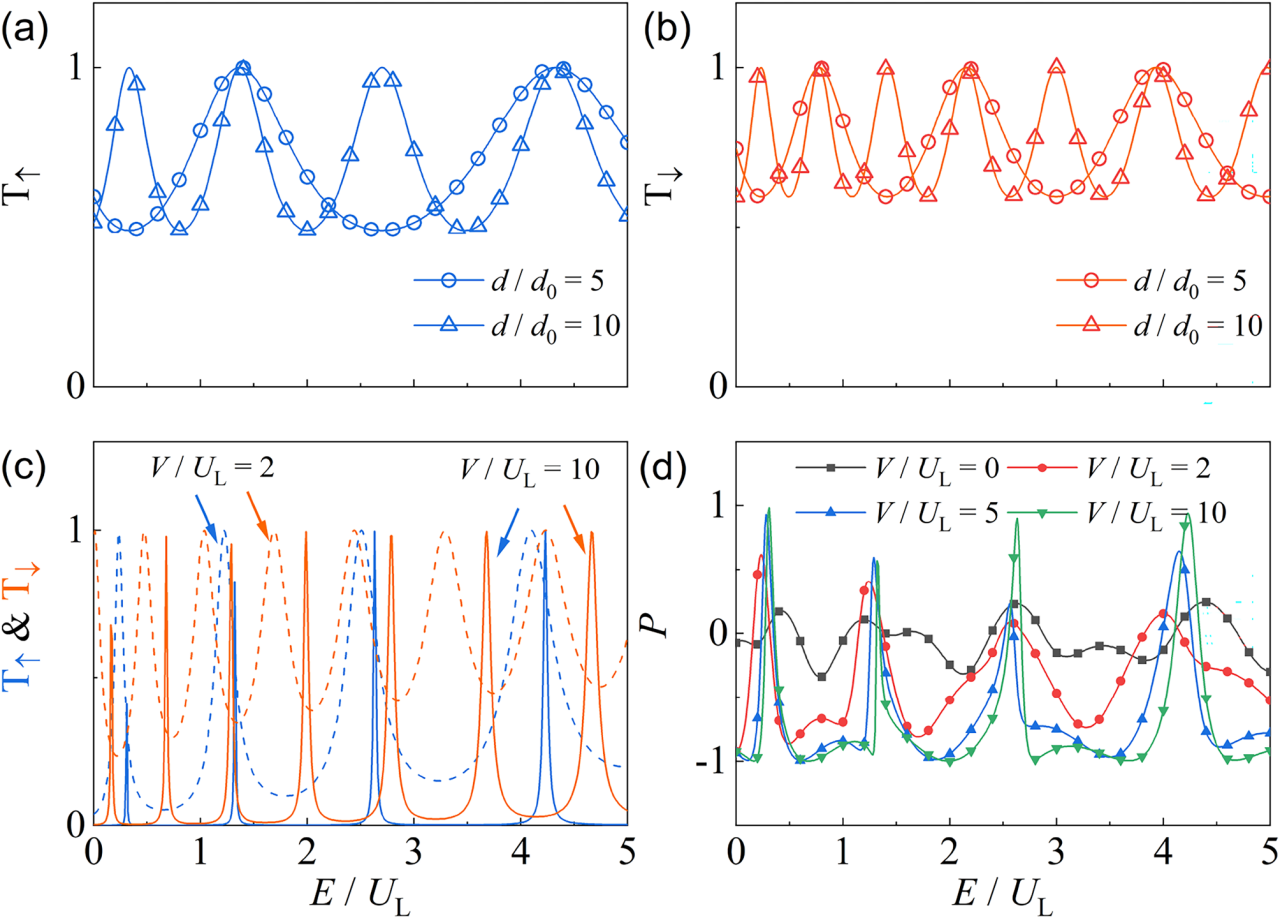
Spin-resolved Fabry–Pérot resonances in the altermagnetic junction at normal incidence ($k_y = 0$). (**a**) and (**b**) Transmission probabilities $T_\uparrow$ and $T_\downarrow$ versus energy for junction lengths $d/d_0 = 5$ and 10. (**c**) Effect of interface barrier $V/U_L$ on $T_\uparrow$ (blue) and $T_\downarrow$ (orange), showing enhanced peak sharpness and spin splitting at higher *V*. (d) Spin polarization *P* versus energy for varying $V/U_L$, demonstrating energy-tunable spin filtering. Parameters: $B=1$, $M=0,5$, $\beta _M=0$ and $U_L=U_g=1$. $V=0$ in (**a**) and (**b**). We set $d_0=\sqrt{B/U_L}$ related to the Fermi wavelength in the leads.

**Fig. 3:**
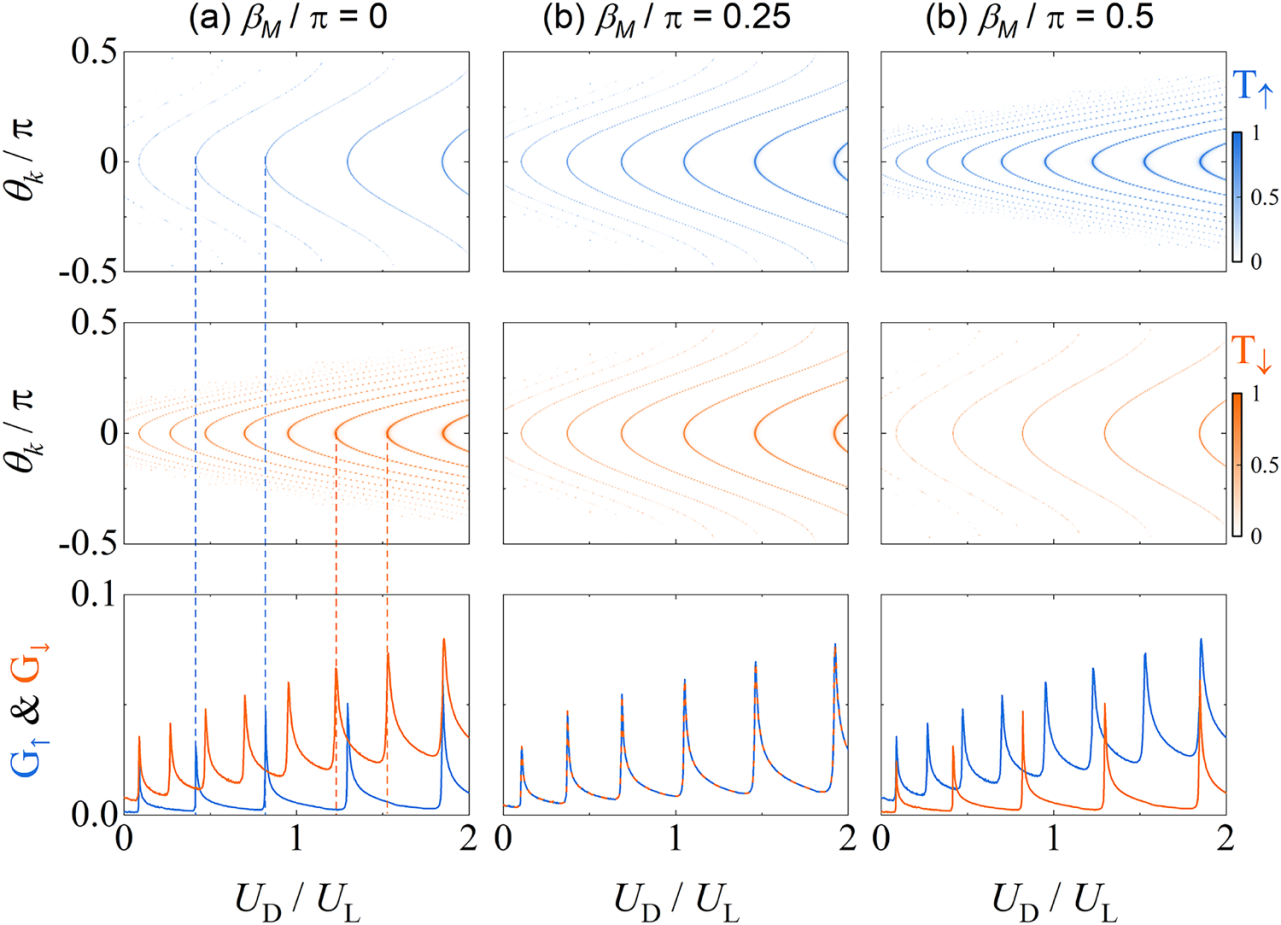
Spin-resolved transmission probability $T_\sigma (\theta _k, U_D)$ (top and middle rows) and conductance $G_\sigma$ (bottom row) for (**a**) $\beta _M = 0$, (**b**) $\beta _M = \pi /4$, and (**c**) $\beta _M = \pi /2$. Vertical dashed lines denote resonance peaks for $\theta _k = 0$ extracted from Fig. 2. The angular asymmetry and spin-resolved conductance oscillations depend sensitively on $\beta _M$, reflecting the anisotropic spin splitting of the altermagnetic junction. Parameters are the same as Fig. 2. Here, we choose $V=5U_L$, $E=0.1U_L$ and $d=20d_0$.

**Fig. 4:**
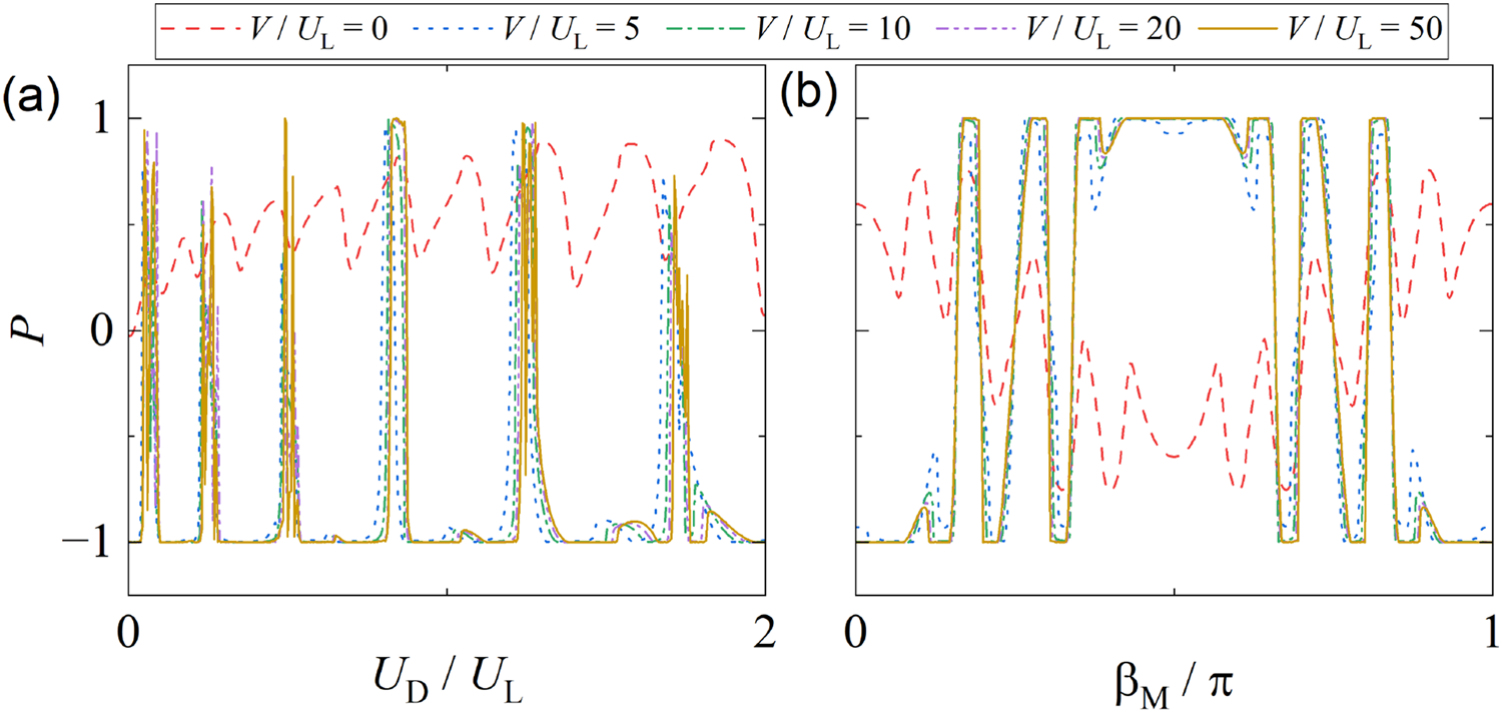
Spin polarization *P* versus (**a**) gate voltage $U_D/U_L$ at fixed $\beta _M=0$ and (**b**) altermagnetic angle $\beta _M/\pi$ at fixed $U_D/U_L=1$, for several barrier strengths $V/U_L$. Sharp plateaus in *P* correspond to spin-selective Fabry–Pérot resonances. Larger *V* enhances spectral selectivity, leading to nearly fully polarized transport. Parameters are the same as Fig. 3.

In this work, we propose a minimal spintronic device comprising a two-dimensional *d*-wave altermagnet embedded between two normal-metal leads [Fig. 1(a)]. We demonstrate that Fabry–Pérot interference [Figs. 2]^79–82^ in this geometry gives rise to spin-resolved transmission resonances that are tunable via gate voltage and the orientation of the altermagnetic order parameter [Figs. 3]. Due to the anisotropic *d*-wave altermagnetic field, spin-up and spin-down electrons accumulate distinct phase shifts, resulting in spin-dependent propagation filtering [Fig. 1(a)]. This mechanism enables energy- and direction-selective transport windows that can be modulated electrically. By solving the spin-resolved quantum scattering problem, we uncover how Fabry–Pérot resonance conditions depend on the internal orientation $\beta _M$ and the electrostatic potential $U_D$, leading to controllable, field-free spin filtering in a simple two-terminal setup [Fig. 4]. Our results identify the *d*-wave altermagnet as a platform for realizing electrically tunable spintronic functionality without magnetic fields or spin–orbit interaction.

This paper is organized as follows. In Sec. 2, we introduce the *d*-wave altermagnetic model and formulate the spin-resolved transport problem. Our main results are presented in Sec. 3, beginning with Sec. 3.1, which illustrates the mechanism of Fabry–Pérot resonances in a pedagogical one-dimensional normal incident case. In Sec. 3.2, we extend the analysis to a two-dimensional geometry, highlighting the roles of junction orientation, gate voltage, and interfacial tunneling in controlling spin-resolved transport. Finally, Sec. 4 summarizes our findings and discusses prospects for implementing gate-tunable spin filters based on altermagnetic materials.

## Model and formalism

### Hamiltonian of the *d*-wave altermagnet

We consider a two-dimensional altermagnet described by a spin-dependent continuum Hamiltonian^18,19^,

1 $$\begin{aligned} \begin{aligned} H_\sigma (\textbf{k}) =\ &B(k_x^2 + k_y^2) \\&+ \sigma M \left[ (k_x^2 - k_y^2) \cos 2\beta _M + 2k_x k_y \sin 2\beta _M \right] , \end{aligned} \end{aligned}$$

where $B = \hbar ^2/2m$ is the inverse effective mass, *M* is the strength of the exchange interaction, and $\beta _M$ denotes the orientation of the altermagnetic order relative to the *x*-axis. The spin index $\sigma = +1$ ($-1$) corresponds to spin aligned (anti-aligned) with the Néel vector, assumed along the *z*-axis. The first term represents an isotropic parabolic band, while the second term captures the momentum-dependent, spin-selective exchange characteristic of altermagnetic order.

To reveal the angular structure of the exchange field, we rewrite Eq. (1) in polar coordinates using $k = \sqrt{k_x^2 + k_y^2}$ and $\phi _k = \arctan (k_y/k_x)$:

2 $$\begin{aligned} H_\sigma (\textbf{k}) = B k^2 + \sigma M k^2 \cos (2\phi _k - 2\beta _M), \end{aligned}$$

which exhibits an effective Zeeman field aligned with the spin quantization axis but modulated by the momentum angle $\phi _k$. This *d*-wave field reaches maxima along $\phi _k = \beta _M$ and $\beta _M \pm \pi /2$, and vanishes at the nodal directions $\phi _k = \beta _M \pm \pi /4$, reflecting the underlying symmetry of the altermagnetic lattice^18,19^.

The corresponding dispersion relation is

3 $$\begin{aligned} \begin{aligned} E_\sigma (\textbf{k}) =\ &B(k_x^2 + k_y^2) \\&+ \sigma M \left[ (k_x^2 - k_y^2) \cos 2\beta _M + 2k_x k_y \sin 2\beta _M \right] , \end{aligned} \end{aligned}$$

yielding spin-split, anisotropic Fermi surfaces. For arbitrary $\beta _M$, these surfaces are elliptical and rotated oppositely for spin-up and spin-down states. Nodes, where spin splitting vanishes, occur along the symmetry-imposed nodal directions discussed above. For example, when $\beta _M = \pi /4$, the nodal lines align with $k_x = \pm k_y$, giving rise to spin-degenerate Fermi crossings.

This behavior stands in contrast to conventional Zeeman splitting, which yields isotropic spin-split bands,

4 $$\begin{aligned} E^Z_\sigma (\textbf{k}) = B(k_x^2 + k_y^2) + \sigma M, \end{aligned}$$

producing concentric Fermi surfaces. In the altermagnet, by contrast, the momentum-dependent spin splitting arises from symmetry-allowed orbital magnetism in the absence of spin–orbit coupling. This nonrelativistic mechanism, recently recognized as generic to certain collinear antiferromagnets, is time-reversal odd and inversion even, allowing spin-split bands that preserve both symmetries.

The anisotropy angle $\beta _M$ plays a pivotal role: it not only governs the direction of maximal spin splitting but also allows for electrical or magnetic control in device settings. It can, in principle, be tuned via external fields, spin injection, or strain engineering, offering a pathway to reconfigurable spintronic functionality.

In the following sections, we explore how this anisotropic band structure leads to rich spin transport phenomena in normal metal–altermagnet–normal metal junctions. We show that the spin-split Fermi surface enables direction- and energy-selective transport, with Fabry–Pérot resonances, spin filtering, and gate-tunable spin polarization controlled by the parameters *d*, $U_g$, *V*, and $\beta _M$.

### Transport formalism of the *d*-wave altermagnetic junction

To investigate spin-resolved quantum transport across an anisotropic altermagnet, we consider a planar junction consisting of a two-dimensional *d*-wave altermagnet sandwiched between two normal metal leads [see Fig. 1]. The system is divided into three regions: region I ($x \le 0$) and region III ($x \ge d$) are spin-degenerate metallic leads, while region II ($0< x < d$) hosts the altermagnetic material. Translational symmetry is preserved along the *y*-direction, so $k_y$ is a conserved quantum number, whereas the *x*-direction requires the substitution $k_x \rightarrow -i\partial _x$ due to the broken translational invariance^79,83^.

The leads are modeled by the spin-independent Hamiltonian

5 $$\begin{aligned} H_{\textrm{I,III}}(-i\partial _x, k_y) = B \left[ (-i\partial _x)^2 + k_y^2 \right] - U_{\text {lead}}, \end{aligned}$$

where $B = \hbar ^2/2m$ is the effective mass coefficient, and $U_{\text {lead}}$ sets the chemical potential in the normal leads. In contrast, the central region is governed by the altermagnetic Hamiltonian incorporating a momentum-anisotropic, spin-selective exchange field:

6 $$\begin{aligned} \begin{aligned} H_{\textrm{II}} =\ &B \left[ (-i\partial _x)^2 + k_y^2 \right] + U_g \\&+ \sigma M \left[ \left( (-i\partial _x)^2 - k_y^2 \right) \cos 2\beta _M + 2(-i\partial _x) k_y \sin 2\beta _M \right] , \end{aligned} \end{aligned}$$

where $U_g$ is a tunable gate potential applied to the altermagnet, and $\sigma = \pm 1$ denotes the spin projection along the Néel vector (assumed *z*-axis). The spin-dependent terms derive directly from the *d*-wave structure analyzed in Sec. IIA.

We solve the stationary scattering problem for a plane wave incident from the left lead. In region I, the wavefunction is given by

7 $$\begin{aligned} \psi _{\textrm{I}}(x) = e^{i k_0 x} e^{i k_y y} + r_\sigma e^{-i k_0 x} e^{i k_y y}, \end{aligned}$$

where $k_0 = k_E \cos \theta _k$ is the longitudinal momentum, with $k_E = \sqrt{(E + U_{\text {lead}})/B}$ and $\theta _k = \arcsin (k_y/k_E)$ defining the incident angle. In region III, the transmitted wave reads

8 $$\begin{aligned} \psi _{\textrm{III}}(x) = t_\sigma e^{i k_0 x} e^{i k_y y}. \end{aligned}$$

In region II, the wavefunction is a linear combination of right- and left-propagating spin-dependent modes,

9 $$\begin{aligned} \psi _{\textrm{II}}(x) = \left[ c_1 e^{i k_+^\sigma x} + c_2 e^{i k_-^\sigma x} \right] e^{i k_y y}, \end{aligned}$$

where the spin-dependent momenta $k_\pm ^\sigma$ are given by

10 $$\begin{aligned} k_\pm ^\sigma = -\sigma \frac{M_s}{B_+^\prime } k_y \pm \sqrt{ \left( \frac{M_s}{B_+^\prime } k_y \right) ^2 + \frac{E - U_g - B_-^\prime k_y^2}{B_+^\prime } }, \end{aligned}$$

with $B_\pm ^\prime = B \pm \sigma M_c$, $M_s = M \sin 2\beta _M$, and $M_c = M \cos 2\beta _M$ quantifying the momentum-dependent and -independent parts of the altermagnetic exchange field, respectively.

At the interfaces $x = 0$ and $x = d$, the boundary conditions include continuity of the wavefunction and discontinuity in its derivative due to interface potentials and spin-selective coupling^45,79,83^:

11 $$\begin{aligned} \psi _{\textrm{I}}(0)&= \psi _{\textrm{II}}(0), \end{aligned}$$

12 $$\begin{aligned} B \left. \frac{d\psi _{\textrm{I}}}{dx} \right| _{x=0} - B_-^\prime \left. \frac{d\psi _{\textrm{II}}}{dx} \right| _{x=0}&= \left( \sigma i M_s k_y - V \right) \psi _{\textrm{I}}(0), \end{aligned}$$

13 $$\begin{aligned} \psi _{\textrm{II}}(d)&= \psi _{\textrm{III}}(d), \end{aligned}$$

14 $$\begin{aligned} B \left. \frac{d\psi _{\textrm{III}}}{dx} \right| _{x=d} - B_-^\prime \left. \frac{d\psi _{\textrm{II}}}{dx} \right| _{x=d}&= \left( \sigma i M_s k_y + V \right) \psi _{\textrm{III}}(d), \end{aligned}$$

where *V* denotes a delta-function barrier at each interface.

Solving the boundary problem yields the spin-dependent transmission amplitude,

15 $$\begin{aligned} t_\sigma (E, \theta _k) = 2 e^{-i d (k_0 - k_+^\sigma - k_-^\sigma )} \frac{B k_0 B_+^\prime (k_+^\sigma - k_-^\sigma )}{\Gamma _+ - \Gamma _-}, \end{aligned}$$

where

16 $$\begin{aligned} \begin{aligned} \Gamma _\pm&= e^{i d k_\mp ^\sigma } \big [ B k_0 \pm (B_-^\prime k_+^\sigma + \sigma M_s k_y) + iV \big ] \\&\quad \times \big [ B k_0 \mp (B_-^\prime k_-^\sigma + \sigma M_s k_y) + iV \big ]. \end{aligned} \end{aligned}$$

The corresponding transmission probability is

17 $$\begin{aligned} T_\sigma (E, \theta _k) = |t_\sigma (E, \theta _k)|^2, \end{aligned}$$

from which the spin-resolved zero-temperature conductance is computed as

18 $$\begin{aligned} G_\sigma (E_F) = G_0 \int _{-\pi /2}^{\pi /2} T_\sigma (E_F, \theta _k) \cos \theta _k \, d\theta _k, \end{aligned}$$

where $G_0 = e^2 W k_F / (2\pi h)$ is the conductance quantum (per spin), and *W* is the transverse width of the junction. The total conductance is the sum^84^

19 $$\begin{aligned} G = G_\uparrow + G_\downarrow , \end{aligned}$$

while the spin polarization of the current is quantified by

20 $$\begin{aligned} P = \frac{G_\uparrow - G_\downarrow }{G_\uparrow + G_\downarrow }. \end{aligned}$$

A value $P = +1$ ($-1$) indicates fully spin-up (spin-down) polarized transport, while $P = 0$ corresponds to an unpolarized current. Hence, *P* serves as a measure of the spin filtering effect arising from the anisotropic band structure of the *d*-wave altermagnet.

## Results and discussion

### Fabry–Pérot resonance in one-dimensional channel

We now analyze the spin-resolved Fabry–Pérot resonance that arises in the altermagnetic junction for electrons incident normally to the interface, i.e., $k_y = 0$. The energy-dependent transmission probabilities for spin-up and spin-down electrons are shown in Figs. 2(a) and (b), respectively. In both cases, the transmission exhibits an oscillatory pattern with energy *E*, which reflects interference due to multiple reflections inside the altermagnetic region. As the junction length increases from $d/d_0 = 5$ to $d/d_0 = 10$, the oscillation period decreases accordingly, as expected from Fabry–Pérot interference^79–82^.

These oscillations reach unity at specific resonance conditions where full transmission is restored. This occurs when the junction length *d* matches an integer multiple of half the spin-dependent wavelength inside the altermagnet. For $k_y = 0$, the resonance condition simplifies to

21 $$\begin{aligned} \kappa _\beta ^\sigma = n\pi /d, \end{aligned}$$

where *n* is an integer, and the wavevector $\kappa _\beta ^\sigma$ satisfies

22 $$\begin{aligned} \kappa _\beta ^\sigma = \sqrt{ \frac{E_n^{\textrm{re}} - U_g}{B + \sigma \delta _\beta M_c} }. \end{aligned}$$

Here, $\delta _\beta = +1$, 0, and $-1$ correspond to $\beta _M = 0$, $\pi /4$, and $\pi /2$, respectively. The resonant energies are therefore given by

23 $$\begin{aligned} E_{n,\sigma }^{\textrm{re}} = U_g + \left( B + \sigma M \right) \left( \frac{n\pi }{d} \right) ^2. \end{aligned}$$

From Eq. (23), we make the following observations: (i) Fabry–Pérot resonances only occur for $E > U_g$, corresponding to propagating modes inside the altermagnet. (ii) For $\beta _M = \pi /4$, the spin dependence vanishes ($\delta _\beta = 0$), and the resonance condition becomes spin-degenerate. This implies that both spin channels exhibit identical resonance peaks, a signature of the $d_{xy}$-wave symmetry, and is consistent with the spin-degenerate nodal directions discussed in Sec. II A. (iii) For $\beta _M \ne \pi /4$, the resonance energies are spin-dependent. In Figs. 2(a) and (b), the spin-up and spin-down transmission peaks are visibly shifted relative to each other, illustrating spin-selective resonances. (iv) The energy spacing between successive resonances is also spin-dependent:

24 $$\begin{aligned} \epsilon _{n,\sigma } = E_{n+1,\sigma }^{\textrm{re}} - E_{n,\sigma }^{\textrm{re}} = \left( B + \sigma M \right) \left( \frac{\pi }{d} \right) ^2 (2n + 1). \end{aligned}$$

For $\beta _M = 0$, this leads to $\epsilon _{n,\uparrow } > \epsilon _{n,\downarrow }$, meaning the spin-down channel supports more closely spaced resonances. This explains the denser oscillation pattern for spin-down electrons in Fig. 2(b).

In off-resonant regions, the transmission is strongly suppressed by the interface barrier potential *V*. This effect is shown in Fig. 2(c), where increasing $V/U_L$ from 2 to 10 dramatically sharpens the resonance peaks and suppresses the background transmission. Due to the spin-dependent resonance conditions, one can tune the incident energy *E* such that it aligns with a transmission peak for one spin species while falling between two peaks for the other. This results in spin-selective transport and gives rise to nearly full spin polarization.

Figure 2(d) illustrates the corresponding spin polarization *P* as a function of energy *E* for various barrier strengths $V/U_L$. For a transparent interface ($V=0$), the polarization remains small and oscillates around zero, indicating equal participation from both spin channels. As *V* increases, regions of nearly full spin polarization ($P \approx \pm 1$) emerge. These occur when the energy is aligned with a resonance peak of one spin species but lies within the off-resonant window of the other.

In particular, for $\beta _M = 0$, $P \approx -1$ appears when $E \sim E_{n,\downarrow }^{\textrm{re}}$ and $E \notin [E_{n,\uparrow }^{\textrm{re}}, E_{n+1,\uparrow }^{\textrm{re}}]$, indicating spin-down-dominated transport. The widths of these polarization plateaus increase at higher energies since $\epsilon _{n,\sigma }$ grows with *n*. Conversely, narrow plateaus with $P \approx +1$ also emerge at low energy when $E \sim E_{n,\uparrow }^{\textrm{re}}$ and the spin-down channel is off-resonant.

These results demonstrate that Fabry–Pérot interference, combined with the anisotropic spin splitting of altermagnets, enables controllable and energy-resolved spin filtering in a simple two-terminal geometry.

### Gate-controlled Fabry–Pérot resonance in two-dimensional altermagnetic junctions

In realistic transport setups, all transverse momentum channels contribute to the conductance, necessitating an angular integration over the incident angle $\theta _k$. In this section, we extend the Fabry–Pérot resonance picture established for normal incidence (Fig. 2) to a fully two-dimensional analysis, incorporating spin-dependent angular filtering and gate-tunable conductance. The central gate $U_D$ in the altermagnetic region serves as a tunable potential barrier, which modulates the resonance condition and enables electric control of spin-selective transport, crucial for implementing spin-polarized current sources in spintronic devices.

Figure 3 (top and middle rows) displays the spin-resolved transmission probability $T_\sigma (\theta _k, U_D)$ as a function of incident angle $\theta _k$ and gate voltage $U_D$, for various orientations $\beta _M$ of the altermagnetic order parameter. For $\beta _M = 0$ [Fig. 3(a)], the spin-up ($\uparrow$) transmission exhibits a broader angular window than the spin-down ($\downarrow$) counterpart, whereas this behavior reverses for $\beta _M = \pi /2$ [Fig. 3(c)]. This anisotropy originates from the spin-dependent longitudinal momentum $k_\pm ^\sigma$ in the central region and determines the critical angle $\theta _c^\sigma$ beyond which the transmission vanishes:

25 $$\begin{aligned} |\theta _k| < \theta _c^\sigma = \arcsin \sqrt{ \frac{U_D - E}{E + U_L} \cdot \frac{B(B + \sigma M_c)}{B^2 - M^2} }. \end{aligned}$$

Here, $M_c = M \cos 2\beta _M$ quantifies the momentum-independent component of the altermagnetic field. For $\beta _M = \pi /4$, $M_c = 0$, yielding $\theta _c^\uparrow = \theta _c^\downarrow$, consistent with the spin-degenerate direction shown in Fig. 3(b). This behavior highlights the role of $\beta _M$ in controlling spin-selective angular filtering.

Summing over all transverse channels, the spin-resolved conductance $G_\sigma$ is obtained from the angular integral in Eq. (22). As shown in the bottom row of Fig. 3, $G_\sigma$ exhibits sharp conductance peaks when the gate voltage $U_D$ satisfies the Fabry–Pérot resonance condition. These peaks correspond to the normal incidence resonant energies discussed previously [dashed lines indicate the $T_\sigma (\theta _k = 0)$ peaks from Fig. 2], and reflect enhanced transmission across a wide angular range. Crucially, at these resonant gate voltages, the conductance is often dominated by a single spin species, enabling gate-tunable spin filtering.

To quantify the net spin transport, we analyze the spin polarization $P = (G_\uparrow - G_\downarrow )/(G_\uparrow + G_\downarrow )$ as a function of $U_D$ and $\beta _M$, shown in Fig. 4. In Fig. 4(a), *P* is plotted versus $U_D/U_L$ for fixed $\beta _M$ and several tunneling barrier strengths $V/U_L$. For a transparent interface ($V = 0$), spin polarization remains low due to poor spectral selectivity. As $V/U_L$ increases, sharp transitions between $P = +1$ and $P = -1$ emerge, indicating fully spin-polarized transport. These transitions are closely tied to the energy separation between spin-resolved resonant levels, as derived in Eq. (24).

Figure 4(b) presents *P* versus $\beta _M/\pi$ at fixed $U_D$, illustrating how the internal altermagnetic orientation modulates spin polarization. For high barrier strengths (e.g., $V/U_L = 50$), *P* develops flat plateaus separated by abrupt jumps, corresponding to spin-resolved Fabry–Pérot resonances aligned with the anisotropic band spin splitting. This behavior demonstrates the role of $\beta _M$ as a band-structure parameter that tunes the geometric phase difference accumulated by spin-up and spin-down quasiparticles in the junction.

There are several remarks for the altermagnets beyond the *d*-wave case studied in the current work: (i) The theoretical formulation of spin-resolved Fabry–Pérot transport can be directly extended to any altermagnet whose electronic structure exhibits distinct Fermi velocities for the two spin subbands. Whenever such a directional spin splitting exists, FP-type interference and spin-polarized conductance naturally emerge, irrespective of the detailed symmetry of the order parameter. (ii) For other even-parity altermagnets, such as *g*- and *i*-wave types^24,55^, although higher-order harmonics in *k* modify the quantitative resonance conditions, spin-polarized transport, tunable via the interfacial barrier strength, remains unchanged qualitatively. (iii) For *g*- and *i*-wave types, the dominant distinction from the *d*-wave case studied in the current worklies in the angular dependence of the polarization on the internal orientation angle $\beta _M$. As illustrated in Fig. 4(b) for the *d*-wave case (period $\pi$), the higher angular harmonics of *g*- and *i*-wave textures imply characteristic periodicities of $\pi /4$ and $\pi /3$, respectively, reflecting their underlying symmetry order.

Altermagnets are characterized by a directional, nonrelativistic spin-splitting Fermi surface accompanied by a vanishing net magnetization^4,19^. This momentum-dependent splitting leads to spin-dependent Fermi velocities and, consequently, spin-dependent Fabry–Pérot resonances in the junction. Because the spin-split Fermi surfaces are locked to the crystallographic orientation, the transport response depends sensitively on the angle between the altermagnetic order and the junction direction, as shown in Fig. 4. This orientation-dependent spin transport in the absence of magnetization is a defining feature of altermagnetism.

By contrast, in ferromagnets, the exchange field is isotropic in momentum space; the two spin bands are uniformly shifted in energy, producing spin-polarized transport that does not vary with orientation. Spin polarization in such systems arises from net magnetization, not from symmetry-protected spin splitting. Altermagnets therefore represent a qualitatively distinct route to spin transport without macroscopic magnetization. In conventional collinear antiferromagnets, combined $\mathcal{P}\mathcal{T}$ symmetry enforces spin degeneracy at each $\boldsymbol{k}$^5^, leading to identical Fermi surfaces for both spin channels. Consequently, spin-dependent resonance and polarization effects are absent in $\mathcal{P}\mathcal{T}$-symmetric antiferromagnets.

In summary, while ferromagnets exhibit spin polarization through uniform magnetization and antiferromagnets remain spin-degenerate, altermagnets uniquely enable symmetry-controlled, spin-polarized transport in the absence of net magnetization.

Taken together, Figs. 3 and 4 confirm that gate voltage $U_D$ and altermagnetic angle $\beta _M$ provide complementary handles to modulate Fabry–Pérot interference and achieve electrically controlled spin filtering. These findings highlight the utility of altermagnets as active elements in spintronic interferometers, offering a route toward gate-tunable spin filters and spin valve devices without requiring a magnetic field or spin-orbit interaction.

As a final remark, the predicted spin-polarized Fabry–Pérot resonances assume a single-domain altermagnetic region across the transport path. Multi-domain averaging would reduce the net polarization, whereas single-domain control, achievable by epitaxial growth, strain engineering, or exchange-bias setting, preserves the symmetry-defined spin orientation required for the effect. For experimental relevance, Table 1 summarizes representative operating windows and feasible device parameters extracted from recent thin-film and ARPES studies.

**Table 1:** Representative operating windows for thin-film altermagnets and device knobs.

Property	RuO$_2$^35,37^	Mn$_5$Si$_3$	CrSb^30,32–34^	Normal-metal leads^85,86^.
$m^*/m_e$	0.5–2	1–3	0.5–2	1
*M* (meV)	$$\lesssim \!\mathcal{O}(10$$–$$100)^{a}$$	10–100	50–200	–
$v_F$ ($10^5$ m/s)	2–6	1–4	2–6	10–15
*Z* (dimensionless)	0–5	0–5	0–4	contact engineered
Feasible $\beta _M$	in-plane 0–$\pi /2$	variant-selectable	epitaxial control	–
Device/knobs	increasing *Z* sharpens FP peaks; $\beta _M$ rotation; gate $U_D$ tunes spin polarization			

Ranges are order-of-magnitude guides extracted from recent thin-film/ARPES/transport studies and typical metal/oxide interfaces. Here *M* denotes the symmetry-allowed nonrelativistic spin-splitting amplitude in the normal state; $Z=V/U_L$ is the dimensionless barrier parameter; $\beta _M$ is the internal orientation relative to the transport axis.

$^{a}$Evidence for symmetry-breaking responses exists; direct altermagnetic spin splitting remains debated.

### Finite-temperature and dephasing effect

The results above are obtained at zero temperature and in the coherent transport limit. For finite temperature, the energy resolution in conductance spectroscopy shown in Fig. 3 and 4 is set by the thermal derivative of the Fermi function, giving a full width at half maximum $\Delta _{\!T}\!\approx \!3.5\,k_B T$^87,88^ thus, FP peak splitting and the associated spin-polarization plateaus remain resolvable provided their intrinsic spacing exceeds $\Delta _{\!T}$. Second, phase-coherent FP contrast decreases with the effective dephasing length, which we denote $L_\textrm{eff}$ to include both phase coherence ($L_\phi$) and spin relaxation ($L_s$). Standard mesoscopic theory and experiment show an approximately exponential suppression of interference visibility $\mathcal {V}$ with junction length *d*, via $\mathcal {V}\!\propto \!\exp [-2d/L_\textrm{eff}]$^89–91^. We therefore state an operating criterion that $d\!\ll \!L_\textrm{eff}$ (empirically, $d/L_\textrm{eff}\!\lesssim \!0.3$–0.5) preserves pronounced FP lineshapes and fully spin polarization plateaus.

To guide experiments, we summarize typical ranges from the literature: micron-scale $L_\phi$ at cryogenic temperatures in clean thin films and topological/oxide platform^92,93^, spin-diffusion lengths $\lambda _s$ of $\sim \!1$–10 nm in heavy metals such as Pt (strong SOC), but $\sim \!0.1$–$3\,\mu$m in high-purity Cu/Au at low *T*^94–96^ and tens of nm up to sub-$\mu$m spin transport reported in antiferromagnets depending on material and orientation^97,98^.

Therefore, in practice, keeping the operating temperature such that $3.5k_BT$ is smaller than the spin-resolved FP level spacing, and designing *d* below the conservative lower bound of $L_\textrm{eff}$ from the chosen material stack, ensures the robustness of both FP lineshapes and the polarization plateaus.

## Conclusion

We have theoretically investigated quantum transport through a two-dimensional junction incorporating a *d*-wave altermagnet with anisotropic, momentum-dependent spin splitting. Using a continuum model, we demonstrated that Fabry–Pérot-type resonances emerge naturally due to coherent multiple scattering within the altermagnetic region. These resonances are highly spin-selective, reflecting the anisotropic spin splitting encoded in the *d*-wave symmetry of the exchange field. In the idealized one-dimensional limit (normal incidence), we derived analytical conditions for resonance energies and showed that the energy spacing and peak positions differ markedly between spin species, depending on the altermagnetic orientation angle $\beta _M$. These spin-resolved Fabry–Pérot resonances give rise to strong energy-dependent spin polarization, particularly in the presence of interfacial barriers that suppress off-resonant transmission. Extending the analysis to two dimensions, we found that both the angular dependence and the spin-resolved conductance are tunable via the gate voltage $U_D$ and the internal altermagnetic orientation $\beta _M$. The critical transmission angle $\theta _c^\sigma$ varies strongly with spin and $\beta _M$, enabling angular spin filtering. We demonstrated that nearly perfect spin polarization ($|P| \approx 1$) can be achieved at resonant gate voltages, with the sign of *P* switchable by tuning $\beta _M$. This behavior is further enhanced by the presence of a central barrier, which sharpens spectral selectivity. Taken together, our results establish *d*-wave altermagnets as a viable platform for electrically controlled spintronic functionalities. By harnessing their intrinsic symmetry-protected spin splitting and interference effects, one can design minimal, all-electric devices such as spin filters, spin valves, and spin logic gates, without requiring external magnetic fields or relativistic spin–orbit coupling. Our findings open pathways for integrating altermagnets into next-generation spintronic architectures, where symmetry engineering and coherent transport play central roles.

## Data Availability

The datasets used and/or analyzed during the current study are available from P.-H. Fu and X.-L. Yu on reasonable request.
